# Supplementary material for: Distinct roles of temporal and frontoparietal cortex in representing actions across vision and language
Source: Nat Commun. 2019 Jan 17;10:289. doi: 10.1038/s41467-018-08084-y (PMC6336825; doi:10.1038/s41467-018-08084-y)
Supplement: Supplementary file 1 — Supplementary Information [file 41467_2018_8084_MOESM1_ESM.pdf]

Distinct roles of temporal and frontoparietal cortex in representing actions across vision and language

Wurm & Caramazza

Supplementary Information

Supplementary Notes

Behavioral performance. During fMRI, participants performed a catch trial detection task. In both the video and sentence session, they detected incorrect actions with good accuracy (sentences: 83% ± 2.2 SEM, videos: 80% ± 2.9 SEM). The rate of false alarms was low for all 8 actions (sentences: 0.9% ± 0.3 SEM, videos: 1.5% ± 0.6 SEM) and uncorrelated between the two sessions ($r(6) = -0.32$, $p = 0.44$) suggesting that there were no action-specific similarities in task difficulty/confusability across sessions. A similar result was obtained in the behavioral control experiment, in which participants performed a 2-alternative forced choice task (Supplementary Table 1).

The Post-fMRI ratings for verbalization and visual action imagery revealed, irrespective of session order, no strong tendencies to verbalize during the video session (mean ratings; sentence first: 3.9 ± 0.5 SEM, video first: 2.7 ± 0.6 SEM; on a Likert scale from 1 to 6) and to imagine the actions during the sentence session (sentence first: 3.1 ± 0.4 SEM, video first: 3.8 ± 0.5 SEM; mixed ANOVA interaction: $F(1,19) = 3.71$, $p = 0.07$). However, the correspondence between verbalized actions and sentences during watching the action videos was stronger when the experiment started with the sentence session as compared to starting with the video session. Likewise, the correspondence between imagined actions and actions shown in the videos was higher when the experiment started with the video session as compared to starting with the sentence session (mixed ANOVA interaction: $F(1,19) = 11.31$, $p = 0.003$). The responses to verbalization/imagery ratings and correspondence ratings correlated with each other, i.e., high verbalization ratings were accompanied by high verbalization-sentence correspondence ratings ($r(19) = 0.45$, $p = 0.035$), and high imagery ratings were accompanied by high imagery-video correspondence ratings ($r(19) = 0.57$, $p = 0.009$), Together, these results suggests only weak tendencies to verbalize and to imagine the action across session; but if participants verbalized or imagined the actions, then the verbalized or imagined actions corresponded more strongly to the stimuli they recalled from the first session.

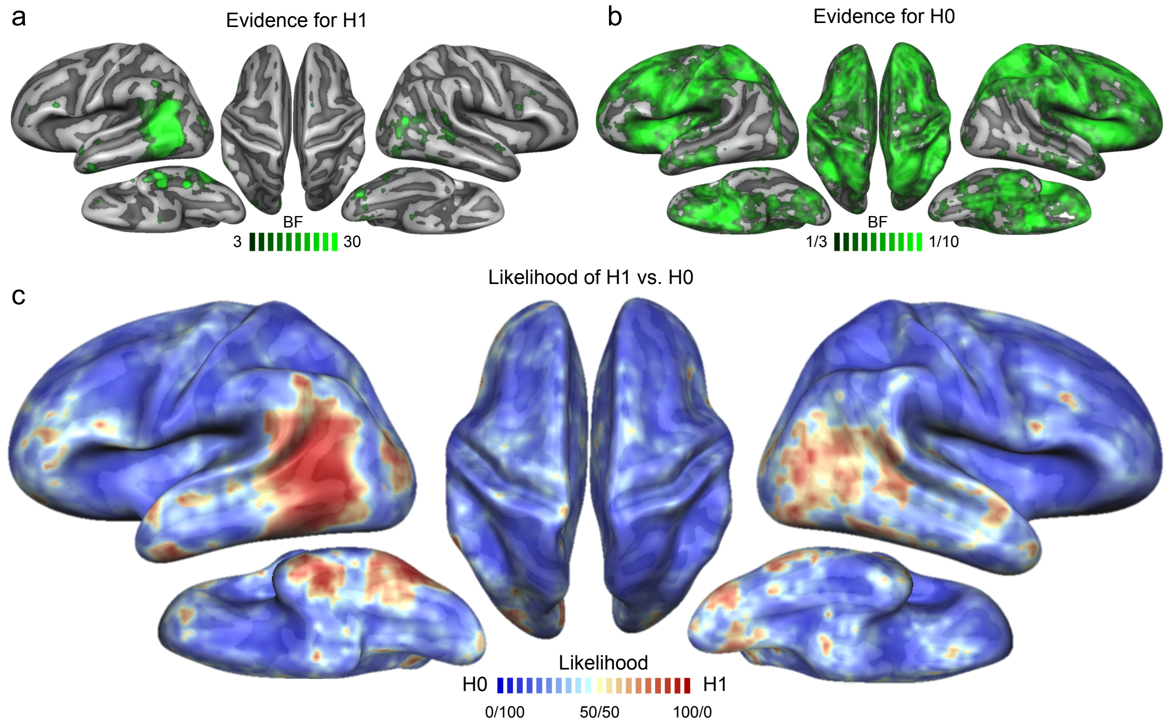

Supplementary Figure 1. Bayesian model comparison for crossmodal action decoding vs. chance. (a) Bayes factors (*BF*) indicating evidence for H_1, i.e., the hypothesis that decoding accuracies are above chance. (b) Inverse Bayes factors indicating evidence for H_0, i.e., the null hypothesis that decoding accuracies are not above chance. Maps are thresholded at BF = 3 and 1/3, suggesting moderate evidence for H_1 and H_0, respectively[1]. Different upper ends of scales for H_1 (30 = very strong evidence) and H_0 (10 = strong evidence) maps were chosen to account for asymmetries in ease to find evidence for H_1 and H_0, respectively. (c) Likelihood map for H_1 vs. H_0using a fixed scale ($BF/(BF+1)*100$). Note that the Bayesian model comparison does not provide statistical measures of significance but likelihoods for or against the tested hypothesis in the data. The general purpose of this analysis is to provide an estimate of whether the absence of crossmodal decoding is meaningful or could be due to a lack of power in the data. Note that the Bayes factor maps are not suited for multiple comparison correction. Therefore, Bayes factors > 3 (and < 100) in right LPTC and left ITG cannot be interpreted as conclusive positive evidence for crossmodal effects in these areas.

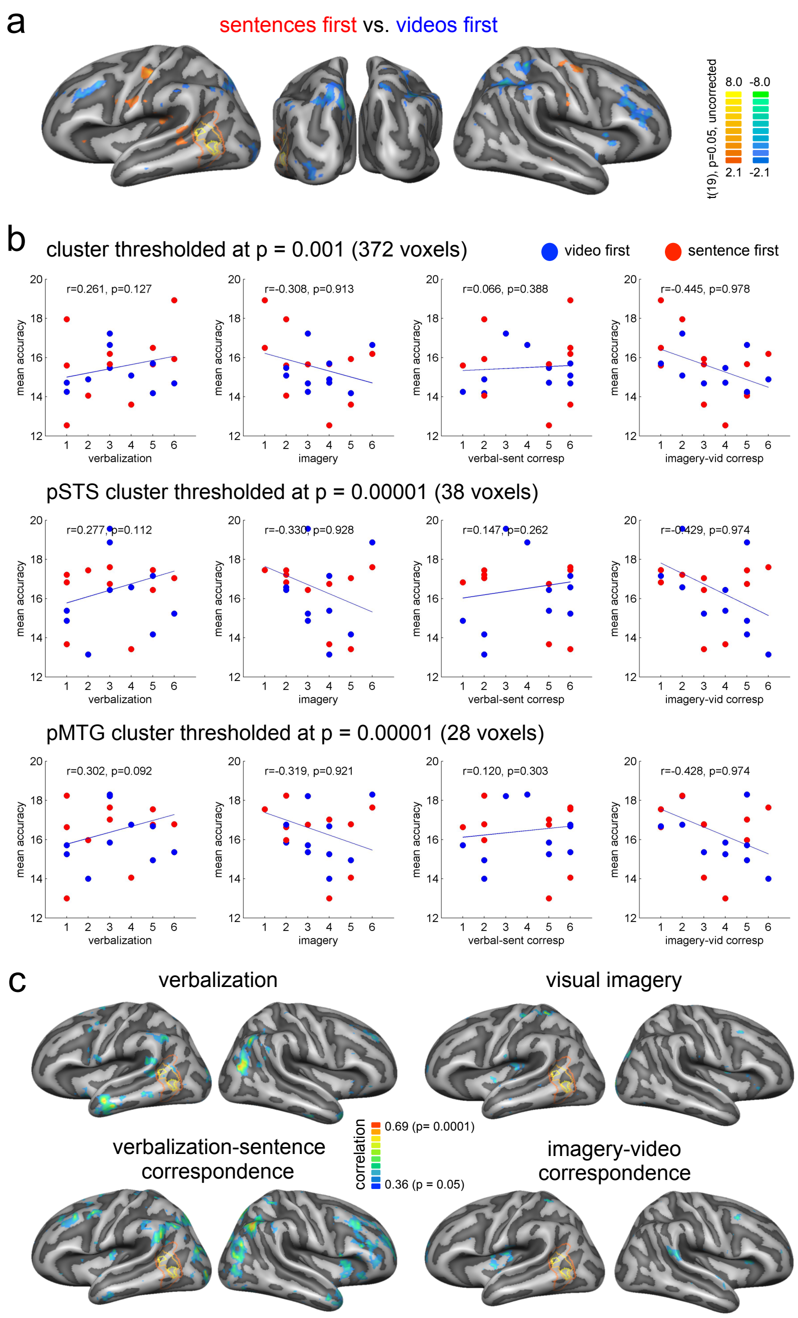

Supplementary Figure 2. Modulation of crossmodal decoding accuracies by verbalization and visual imagery. (a) Independent two-tailed t-test between decoding accuracies of the participant group with session order sentences-videos (sentence first) and the participant group with session order videos-sentences (video first). To reveal any trends of session order effects, maps were leniently thresholded and uncorrected. (b) Correlations between decoding accuracies and rating scores for verbalization, visual imagery, correspondence between verbalized actions and sentences, and correspondence between imagined actions and videos (see Methods for details). Decoding accuracies were averaged across voxels that showed significant crossmodal decoding. As the cluster of the crossmodal decoding was relatively large (372 voxels) it could be that a true effect emerging from a subset of voxels in the cluster was averaged out. We therefore tested whether reducing the ROI size by including only the most significant voxels (p < 0.0001; 38 voxels in pMTG, 28 voxels in pSTS). Blue dots indicate participants of the “video first” group, red dots indicate participants of the “sentence first” group. (c) Whole brain maps of correlations between rating scores and crossmodal decoding accuracies. Outlines in a and c indicate the extent of the crossmodal action decoding cluster thresholded at p < 0.001 (red), p < 0.0001 (orange), and p < 0.00001 (yellow).

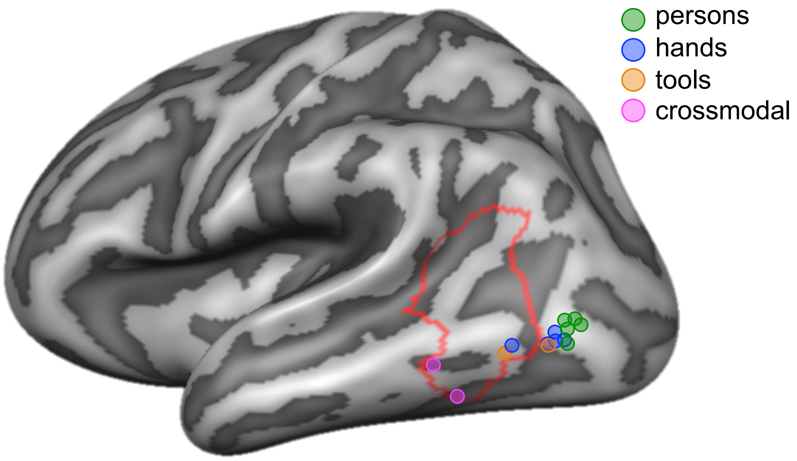

Study	Contrast	Tal X	Tal Y	Tal Z
Bracci et al., 2012	tools > animals (Study 1)	-48	-65	-6
	hands > animals (Study 1)	-49	-65	-2
	hands > chairs (Study 2)	-46	-68	-2
	tools > chairs (Study 2)	-46	-68	-2
	whole bodies > chairs (Study 2)	-46	-73	-2
Bracci & Peelen, 2013	hands > whole bodies + chairs (Study 1)	-50	-71	-3
	hands > whole bodies + chairs (Study 2)	-48	-69	1
	hands > whole bodies + chairs (Study 3)	-47	-69	1
	hands > whole bodies + chairs (Study 4)	-49	-69	-2
	whole bodies > hands + chairs (Study 1)	-47	-72	3
	whole bodies > hands + chairs (Study 2)	-46	-77	7
	whole bodies > hands + chairs (Study 3)	-45	-74	5
	whole bodies > hands + chairs (Study 4)	-45	-74	6
Bracci et al., 2015	whole bodies > chairs	-48	-71	5
Fairhall & Caramazza, 2013	crossmodal MVPA	-50	-48	-7
Simanova et al., 2012	crossmodal MVPA	-43	-52	-7

Supplementary Figure 3. Peak locations of functional localizers for objects. Talairach coordinates were extracted from 3 studies that report univariate contrasts localizing object categories that broadly comprise the objects targeted in the present study (whole bodies: green, hands: blue, and tools: orange)[2-4]. Additional Talairach coordinates (converted from MNI coordinates) were extracted from 2 studies targeting modality-general representations of object categories using crossmodal MVPA (Fairhall & Caramazza, 2013: tools, fruit, clothes, mammals, and birds; Simanova et al., 2012: tools and animals)[5,6]. Peak locations do not substantially overlap with the cluster for crossmodal action decoding of the present study (red outline).

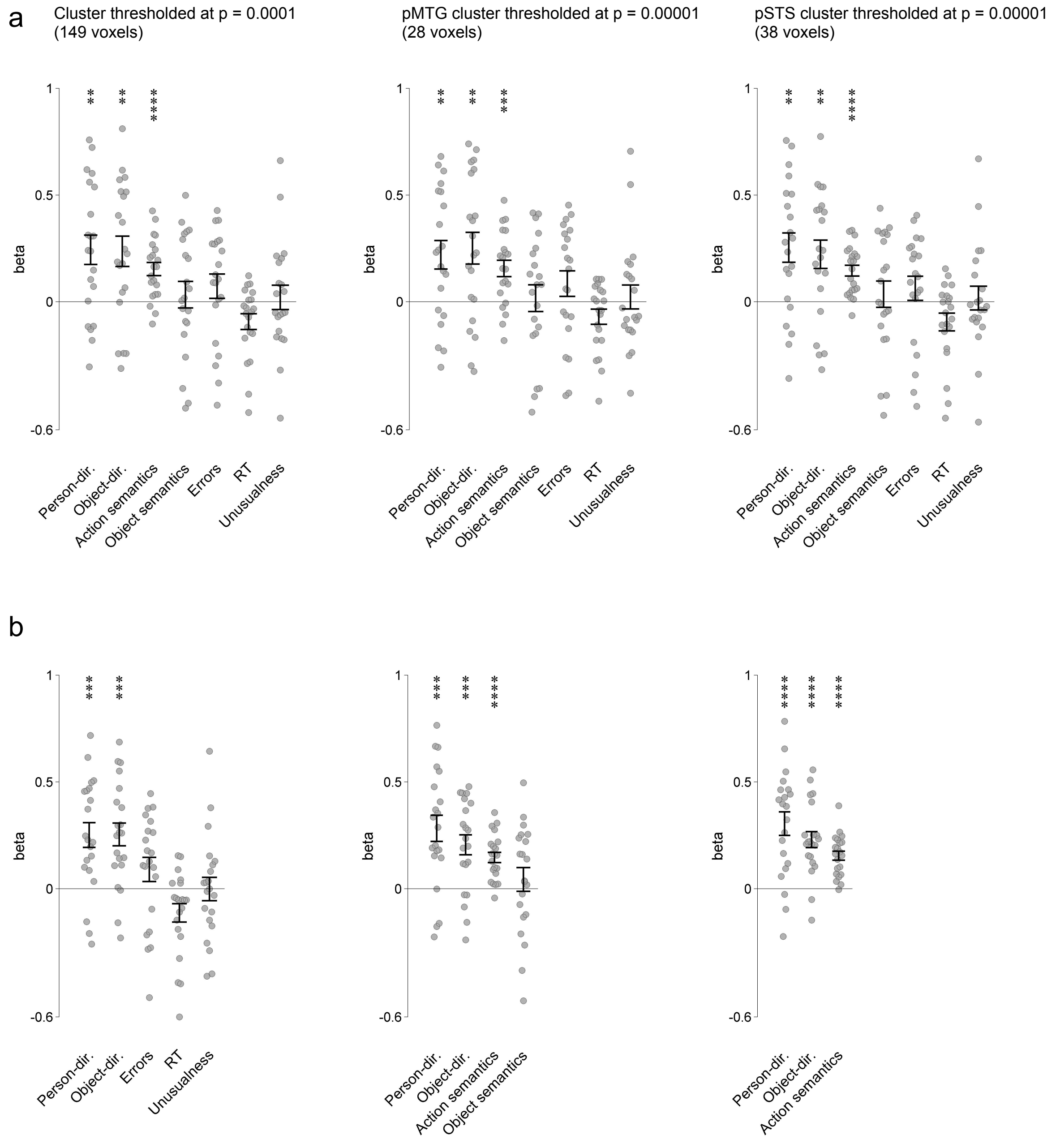

Supplementary Figure 4. Robustness of crossmodal multiple regression RSA effects across different ROI sizes (a) and models used in the multiple regression RSA (b). Asterisks indicate FDR-corrected effects: * $p < 0.05$, ** $p < 0.01$ *** $p = 0.001$, **** $p < 0.0001$. Error bars/lines indicate SEM.

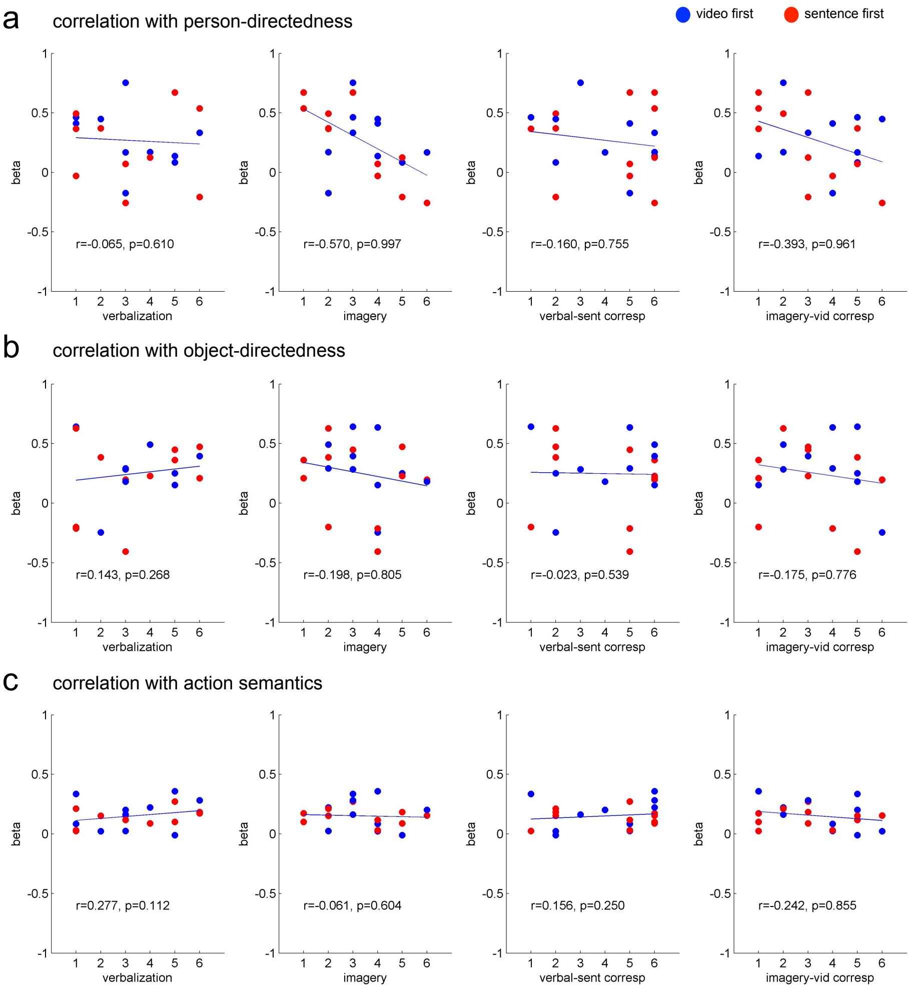

Supplementary Figure 5. Modulation of beta coefficients from the crossmodal multiple regression RSA (Fig. 3b) by verbalization and visual imagery. Betas for person-directedness (a), object-directedness (b), and action semantics (c) were correlated with rating scores for verbalization, visual imagery, correspondence between verbalized actions and sentences, and correspondence between imagined actions and videos (same procedures as reported for Supplementary Figure 2). We observed a trend for a negative correlation between person-directedness and imagery (when using a two-tailed test). However, this effect was not significant after FDR correction for the number of correlations, suggesting that the negative correlation was likely to arise by chance. We additionally tested for effects of session order by entering the betas for person-directedness, object-directedness, and action semantics into two-tailed independent *t*-tests comparing the video-first with the sentence-first group. No significant differences between groups were observed (all *p*s > 0.1).

Supplementary Table 1. Behavioral results of the fMRI experiment, the behavioral (two-alternatives forced choice) control experiment, and the rating (using Likert scales from 1 = not at all to 6 = very much; see Methods for details).

	open	close	give	take	stroke	scratch	agree	disagree	catch trials
fMRI sentence session									
mean hit/CR rate	0.990	0.992	0.973	0.999	0.992	0.994	0.995	0.996	0.832
SEM	0.003	0.003	0.005	0.001	0.003	0.003	0.003	0.003	0.022
fMRI video session									
mean hit/CR rate	0.988	0.981	0.998	0.991	0.959	0.987	0.993	0.978	0.805
SEM	0.003	0.008	0.001	0.004	0.016	0.006	0.004	0.012	0.029
2AFC sentence session									
mean accuracy	0.986	0.993	0.972	0.979	0.979	0.993	1.000	1.000	0.819
SEM	0.009	0.007	0.021	0.011	0.015	0.007	0.000	0.000	0.056
mean RT	1961	1968	2044	1963	1973	1954	1986	1972	2121
SEM	91	98	106	100	83	101	77	78	73
2AFC video session									
mean accuracy	0.986	1.000	1.000	1.000	0.924	0.972	0.993	1.000	0.875
SEM	0.009	0.000	0.000	0.000	0.024	0.012	0.007	0.000	0.035
mean RT	1923	1903	1819	1821	1753	1689	1670	1657	1897
SEM	66	69	71	67	93	90	85	80	70
unusualness sentences									
mean rating	1.09	1.36	1.18	1.09	2.09	1.18	1.27	1.45	NA
SEM	0.09	0.28	0.12	0.09	0.37	0.12	0.19	0.25	NA
unusualness videos									
mean rating	1.18	1.00	1.36	1.36	2.18	1.45	1.91	2.00	NA
SEM	0.12	0.00	0.20	0.28	0.44	0.28	0.31	0.36	NA
Person-directedness									
mean rating	1.15	1.25	4.20	3.40	1.20	1.15	5.50	5.65	NA
SEM	0.08	0.12	0.39	0.41	0.12	0.11	0.24	0.17	NA
Object-directedness									
mean rating	5.80	5.80	5.65	5.75	1.65	1.70	1.15	1.15	NA
SEM	0.09	0.12	0.17	0.12	0.32	0.35	0.11	0.11	NA

Supplementary Table 2. One-tailed t-tests and Bayesian comparisons for within-video, within-sentence, and crossmodal decoding.

	within-video			within-sentence			crossmodal		
	t(20)	*p*	*BF*	*t*(20)	*p*	*BF*	*t*(20)	*p*	*BF*
LPTC	11.18	<0.0001	>1000	5.10	<0.0001	953.27	4.17	0.0002	138.61
IPS	8.56	<0.0001	>1000	5.64	<0.0001	>1000	-1.20	0.8773	0.11
PMC	7.33	<0.0001	>1000	2.78	0.0058	8.88	-0.85	0.7984	0.13
IFG	5.55	<0.0001	>1000	3.91	0.0004	81.31	-0.08	0.5314	0.21

Supplementary References

1. Jeffreys H. *The theory of probability*. OUP Oxford (1998).
2. Bracci S, Caramazza A, Peelen MV. Representational Similarity of Body Parts in Human Occipitotemporal Cortex. *J Neurosci* **35**, 12977-12985 (2015).
3. Bracci S, Cavina-Pratesi C, Ietswaart M, Caramazza A, Peelen MV. Closely overlapping responses to tools and hands in left lateral occipitotemporal cortex. *J Neurophysiol* **107**, 1443-1456 (2012).
4. Bracci S, Peelen MV. Body and object effectors: the organization of object representations in high-level visual cortex reflects body-object interactions. *J Neurosci* **33**, 18247-18258 (2013).
5. Fairhall SL, Caramazza A. Brain regions that represent amodal conceptual knowledge. *J Neurosci* **33**, 10552-10558 (2013).
6. Simanova I, Hagoort P, Oostenveld R, van Gerven MA. Modality-independent decoding of semantic information from the human brain. *Cereb Cortex* **24**, 426-434 (2014).
